# BiGSM: Bayesian inference of gene regulatory network via sparse modelling

**DOI:** 10.1093/bioinformatics/btaf318

**Published:** 2025-06-09

**Authors:** Hang Qin, Mateusz Garbulowski, Erik L L Sonnhammer, Saikat Chatterjee

**Affiliations:** Digital Futures, and School of Electrical Engineering and Computer Science, KTH Royal Institute of Technology, Stockholm 11428, Sweden; Department of Biochemistry and Biophysics, Stockholm University, Science for Life Laboratory, Solna 17121, Sweden; Department of Immunology, Genetics and Pathology, Uppsala University, Uppsala 75123, Sweden; Department of Biochemistry and Biophysics, Stockholm University, Science for Life Laboratory, Solna 17121, Sweden; Digital Futures, and School of Electrical Engineering and Computer Science, KTH Royal Institute of Technology, Stockholm 11428, Sweden

## Abstract

**Motivation:**

Inference of gene regulatory network (GRN) is challenging due to the inherent sparsity of the GRN matrix and noisy expression data, often leading to a high possibility of false positive or negative predictions. To address this, it is essential to leverage the sparsity of the GRN matrix and develop a robust method capable of handling varying levels of noise in the data. Moreover, most existing GRN inference methods produce only fixed point estimates, which lack the flexibility and informativeness for comprehensive network analysis. In contrast, a Bayesian approach that yields closed-form posterior distributions allows probabilistic link selection, offering insights into the statistical confidence of each possible link. Consequently, it is important to engineer a Bayesian GRN inference method and rigorously execute a benchmark evaluation compared to state-of-the-art methods.

**Results:**

We propose a method—Bayesian inference of GRN via Sparse Modelling (BiGSM). BiGSM effectively exploits the sparsity of the GRN matrix and infers the posterior distributions of GRN links from noisy expression data by using the maximum likelihood based learning. We thoroughly benchmarked BiGSM using biological and simulated datasets including GeneNetWeaver, GeneSPIDER, and GRNbenchmark. The benchmark test evaluates its accuracy and robustness across varying noise levels and data models. Using point-estimate based performance measures, BiGSM provides an overall best performance in comparison with several state-of-the-art methods including GENIE3, LASSO, LSCON, and Zscore. Additionally, BiGSM is the only method in the set of competing methods that provides posteriors for the GRN weights, helping to decipher confidence across predictions.

**Availability and implementation:**

Code implemented via MATLAB and Python are available at Github: https://github.com/SachLab/BiGSM and archived at zenodo.

## 1 Introduction

The interactions among genes or proteins often occur when they are functionally related (Boone *et al.* 2007). Such interactions from the basis of biological regulations have been extensively studied and further characterized by various experimental techniques (Sharma *et al.* 2016, Hafner *et al.* 2021). Importantly, advancements in current high-throughput sequencing techniques have revolutionized our ability to measure gene expression levels in biological systems, enabling researchers to infer gene regulatory networks (GRNs). In essence, a GRN captures the activatory or inhibitory interactions between genes, based on their expression-based co-dependence. These regulatory mechanisms are especially critical in the context of cancer cells, where mutated genes may exhibit enhanced or suppressed effects on cellular functions (Ashworth *et al.* 2011). By understanding these complex interactions, scientists can uncover the underlying regulatory architecture that drives disease progression. For example, inferring a regulome in cancer or other complex diseases can significantly aid in the design of personalized treatment strategies for patients. Specific studies have demonstrated the utility of GRN inference in elucidating regulatory networks of binding elements in lung cancer (Liang *et al.* 2020) and unravelling the regulatory mechanisms related to the MYC pathway in castration-resistant prostate cancer (Panja *et al.* 2024). One of the biggest challenges in GRN inference is accurately predicting a network that minimizes false positive links. This challenge is closely related to the sparsity of the GRN matrix. Since the true GRN matrices are usually very sparse, achieving a similar level of density in the predicted GRN matrix helps reduce the number of false positive links.

Over the years, a wide spectrum of GRN inference methods have been developed, including score-based methods, regression (estimation)-based methods, and methods that use recent advances in deep learning, e.g. DeepSEM (Shu *et al.* 2021). Score-based methods use specially designed scores (features) to indicate the inferred importance of potential regulations, for example, GENIE3 (Huynh-Thu *et al.* 2010) uses weights of decision trees, TIGRESS (Haury *et al.* 2012) based on least angle regression (LARS), while CN (Aghdam *et al.* 2015), OIPCQ (Mahmoodi *et al.* 2021), and PCA-CMI (Zhang *et al.* 2012) are based on Mutual Information (MI). Methods that directly estimate the GRN matrix by solving a specially designed GRN model are regression (estimation)-based, such as LSCON (Hillerton *et al.* 2022) and LASSO (Tjärnberg *et al.* 2015). In addition, several toolboxes facilitate comprehensive analysis and inference of GRNs, such as GeneSPIDER (Tjärnberg *et al.* 2017, Garbulowski *et al.* 2024) or Network ZOO (Ben Guebila *et al.* 2023). While LASSO uses sparsity explicitly, a majority of these methods do not use the sparsity knowledge efficiently.

Importantly, to ensure accurate predictions, various assumptions should be met in GRN inference. For example, knowledge about gene perturbation is crucial for obtaining an accurate GRN (Seçilmiş *et al.* 2022b). One of the pitfalls in this field is the high chance of inferring false positive links from noisy data. Therefore, it is imperative to develop GRN inference methods that effectively handle noisy data. This can be accomplished by making reasonable assumptions on how perturbations and noise affect gene expression data. Furthermore, instead of producing a fixed inferred GRN, employing a Bayesian framework that provides a complete inferred posterior distribution of links is more informative. However, there are currently few Bayesian frameworks available for GRN prediction. One notable example is GRNVBEM (Sanchez-Castillo *et al.* 2018), which infers GRN from time series and pseudo-time series data using an autoregressive moving-average model within a variational Bayesian framework. Despite its strengths, GRNVBEM has limitations: it can only work on time-labelled data and does not use the sparsity property of GRNs; the perturbation design is not included in the system, which is essential for accurate GRN inference. Addressing these gaps is critical for advancing the field and improving the reliability of GRN predictions.

To tackle these challenges, we developed a novel approach to GRN inference inspired by Sparse Bayesian Learning (SBL) methods, such as Relevance Vector Machine (RVM) (Tipping, 1999, 2001), SBL for basis selection (Wipf and Rao 2004), and Bayesian compressive sensing (BCS) (Ji *et al.* 2008). RVM and BCS aim to estimate a sparse signal from its noisy linear measurement, aligning perfectly with the sparse nature of GRN matrices. A previous attempt was made to apply SBL to GRN inference (Chan *et al.* 2007), however, this work did not include perturbation in the GRN model and only has been tested on a small network with 16 genes. Besides, this method assumed the time-series expression data as an AR-1 model and the GRN matrix as a transition matrix, which is not a reasonable assumption. None of the SBL methods were rigorously evaluated using benchmark datasets and compared against state-of-the-art methods. To this end, we proposed a novel GRN inference method called Bayesian inference of GRN via Sparse Modelling (BiGSM), and evaluated it using several well-known benchmark datasets. The BiGSM method allows us to infer the posterior distribution of GRNs from noisy gene expression data under the known perturbation.

We benchmarked the BiGSM method against several inference methods including LSCON (Hillerton *et al.* 2022), LASSO (Tjärnberg *et al.* 2015), SVM, Zscore (Prill *et al.* 2010), and GENIE3 (Huynh-Thu *et al.* 2010). The experiments used the GeneSPIDER toolbox (Tjärnberg *et al.* 2017, Garbulowski *et al.* 2024), DREAM (Dialogue for Reverse Engineering Assessment and Methods) (Stolovitzky *et al.* 2007) collections from GeneNetWeaver (GNW) (Schaffter *et al.* 2011), the GRNbenchmark webserver (Seçilmiş *et al.* 2022a), and an additional biological network of the SOS path way in *Escherichia coli* (Gardner *et al.* 2003). In GeneSPIDER, most inference methods rely on technical replicates, making it important to have at least two replicates. However, we demonstrate that BiGSM is highly accurate in inferring GRNs even with noisy data where only one replicate is available. We also analysed the inferred posterior distributions, highlighting their relationship to accurately inferred GRN links. Additionally, we compared the densities of the inferred GRN matrices with those generated by other inference methods. Thanks to its sparse modelling capabilities, BiGSM predictions most accurately match the densities of real GRN matrices.

## 2 Methodology

### 2.1 Datasets

The performance of BiGSM is mainly evaluated on the GeneSPIDER toolbox, the GRNbenchmark web server, collections of DREAM challenge datasets, and *E. coli*. biological data Gardner *et al.* (2003). A summary of the datasets used is given in Table 1, available as supplementary data at *Bioinformatics* online. GeneSPIDER can generate synthesis networks that resemble biological GRNs with a scale-free topology. We set the sparsity of networks to an average of three links per gene, reflecting the typical sparsity level observed in most biological GRNs. To demonstrate that BiGSM is consistently reliable across varying noise levels, we generated test data with different SNRs of 1, 0.1, and 0.01. Additionally, GeneSPIDER can simulate different perturbation experiments by configuring a perturbation matrix. For our study, we used single-gene knockdown perturbations for each gene in a network. Fold changes were directly simulated in GeneSPIDER expression data. The SNR for GeneSPIDER data was defined as


(1) $$\text{SNR}=\frac{\Sigma_{\text{min}}(Y)}{\sqrt{\chi^{-2}(\alpha ,N,M)\lambda }},$$

where $\Sigma_{\text{min}}$ is the smallest singular value of the measured gene expression matrix Y, λ is the variance of noise, and $\chi^{-2}(\alpha ,N,M)$ is the Chi-Square distribution with N×M degrees of freedom. *N* and *M* are the number of genes and experiments.

GRNbenchmark.org is a webserver for benchmarking GRN inference methods. It offers users an integrated suite of benchmarks across various datasets including GeneNetWeaver and GeneSPIDER. Each dataset spans a range of properties, including multiple noise levels to simulate different experimental conditions. After the benchmarking is completed, the server provides evaluation results through interactive summary plots and detailed underlying curves. We selected GRNbenchmark for testing to ensure a fair and transparent evaluation of our method.

The DREAM challenge datasets include both in silico networks generated with GeneNetWeaver (GNW) and in vivo networks. For our evaluation, we specifically selected the following datasets: DREAM3 In Silico Size 50 Knockout dataset, DREAM4 In Silico Size 100 Knockdown dataset, and DREAM5 In Silico, *E. coli*. datasets. For the DREAM5 dataset, since the expression data is provided in chip features that encompass multiple experiments, we focused on subnetworks according to chip features files and only retained the knockdown and overexpression experiments. Although the DREAM dataset is widely used, the data generation model used by GNW does not incorporate perturbations as a matrix input. Therefore, the GNW and DREAM datasets do not provide an explicit perturbation matrix. Since the knowledge of perturbation design has been shown to be important for the accuracy of GRN inferences (Seçilmiş *et al.* 2022b), using the DREAM and GNW dataset alongside GeneSPIDER may introduce model mismatch issues, potentially leading to variations in inference performance.

### 2.2 Bayesian inference of gene regulatory network

In this section, we introduce our algorithm called Bayesian inference of GRN via sparse modelling (BiGSM). For a GRN with N genes, we define the adjacency matrix as $A\in R^{N\times N}$, where the element at the *i*th row and the *j*th column $A_{ij}$ is the weighted regulation from a regulator gene j to target gene *i*. The perturbation matrix is denoted as P, and the measured fold change in gene expression is represented by Y. Considering replications in the experiments, the expression matrix is $Y\in R^{N\times rN}$, where *r* is the number of technical replications.

Assuming the perturbations around steady state, the dynamics of the network can be projected onto a general linear mapping model (Tegner *et al.* 2003). Therefore, the nonlinear system modelling gene expression and GRN is approximated using a first-order differential equation (Gardner *et al.* 2003)


(2) $$\frac{d\tilde{y}}{dt}=A\tilde{y}+\tilde{p},$$

where $\tilde{y}$ is a column vector representing gene expression of *N* genes; $\frac{d\tilde{y}}{dt}$ represents the rate of accumulation of the species in $\tilde{y}$, and $\tilde{p}$ is a column vector of external perturbation. Under the steady-state assumption ($\frac{d\tilde{y}}{dt}=0$), the equation above reduces to $\tilde{y}=-A^{-1}\tilde{p}$. If an individual perturbation is implemented on each of the *N* genes in A, we can stack *N* column vectors of $\tilde{p}$ and $\tilde{y}$ and obtain matrix Y and P. Considering the noisy measurement, we have the following equation


(3) $$Y=-A^{-1}P+E,$$

where E represents the additive measurement noise matrix affecting the measurements.

Driven by the system assumption above, the inference of GRN can be described as inferring matrix A from a given Y and P. To simplify the computation, our BiGSM performs a row-by-row inference. The equation can be rewritten as $p_{i}=-Y^{⊤}a_{i}+E^{⊤}a_{i}$, where $p_{i}$ and $a_{i}$ are the $i^{th}$ row of P and A. Considering only a row vector a of the GRN matrix A and one row vector p of the perturbation matrix P, further simplifying the noise term $E^{⊤}a_{i}$ as e and define $H=-Y^{⊤}$, the equation becomes


(4) p=Ha+e.

BiGSM provides Bayesian inference with sparsity modelling. Compared to point estimation methods such as Least Square Cut Off (LSCO) and Least Absolute Shrinkage and Selection Operator (LASSO), BiGSM gives an individual posterior distribution for every link in GRN. Since the GRN matrix A is usually very sparse, utilizing the sparsity allows reducing false positive predicted links and leads to an accurate inference. To achieve this, we first define a zero-mean Gaussian prior distribution to each link inside a,


(5) $$f(a|\alpha )=N(a|0,\mathrm{diag}{\left(\alpha \right)}^{-1}))=N(a|0,\Lambda^{-1})),$$

where $\alpha =(\alpha_{1},\alpha_{2},\ldots ,\alpha_{n},\ldots ,\alpha_{N})$, $\alpha_{n}$ is the precision of a Gaussian density function of $a_{n}$. $a=(a_{1},a_{2},\ldots ,a_{n},\ldots ,a_{N})$. This step initialized each possible link in a GRN as a Gaussian random variable with zero mean and initial variance. During the subsequent learning process, the precision vector α will be continuously updated. We assumed the noise vector e is an i.i.d zero-mean Gaussian noise, the noise distribution is


(6) $$f(e|\beta )=N(e|0,\beta^{-1}I),$$

where β is the precision of noise and $I_{N}$ is an identity matrix of size *N*. The likelihood is


(7) $$f(p|H,a,\beta )=N(p|\mathrm{Ha},\beta^{-1}I_{N}).$$

From the Bayesian rule, the posterior distribution of a is


(8) $$\begin{array}{l} f\left(a|p,H,\alpha ,\beta \right)=N(a|\mu ,\Sigma )\\ \mu =\beta \Sigma (H^{⊤}p)\\ \Sigma ={\left(\Lambda +\beta H^{⊤}H\right)}^{-1}. \end{array}$$

This posterior distribution provides the inferred probability distributions for every possible link in the GRN. The inferred GRN can be estimated as a point estimate by taking the mean values of these distributions. Initially, we assume that all links do not exist by setting a zero-mean Gaussian prior, reflecting the sparse nature of the GRN matrix where most entries are zero. Bayesian inference then updates this prior by incorporating the observed expression data H and perturbation design p, yielding the posterior distribution. Since most links in the GRN matrix are indeed zero, the Bayesian inference can more effectively identify these non-existent links. To learn accurate posterior distributions, a type-2 Maximum Likelihood (ML) learning is used by integrating over α and taking the logarithm to find the marginal likelihood and log-likelihood:


(9) $$\begin{array}{l} f(p|H,\alpha ,\beta )=\int f(p|H,a,\beta )f(a|\alpha )da\\ =N(p|0,\beta^{-1}I_{N}+H\Lambda^{-1}H^{⊤}). \end{array}$$

And the log likelihood is


(10) $$\begin{array}{l} L(H,\alpha ,\beta )=\text{log}\;f(p|H,\alpha ,\beta )\\ =-\frac{1}{2}N\ln(2\pi )-\frac{1}{2}\text{log}\;|\beta^{-1}I_{N}+H\Lambda^{-1}H^{⊤}|\\ -\frac{1}{2}p^{⊤}{\left(\beta^{-1}I_{N}+H\Lambda^{-1}H^{⊤}\right)}^{-1}p. \end{array}$$

The maximum likelihood learning determines the optimal α and β. By setting up $\frac{\partial L}{\partial \alpha }=0$ and $\frac{\partial L}{\partial \beta }=0$, we derive the following updating formulas for the prior and noise precision,


(11) $$\begin{array}{l} \alpha_{i}^{\mathrm{new}}=\frac{1-\alpha_{i}\Sigma_{ii}}{\mu_{i}^{2}},\\ \frac{1}{\beta^{\mathrm{new}}}=\frac{\|p-H\mu {\|}_{2}^{2}}{\dim(A,0)-\sum_{i}\left(1-\alpha_{i}\Sigma_{ii}\right)}, \end{array}$$

where $\alpha_{i}$ is the $i^{th}$ element in α and $\Sigma_{ii}$ is the $i^{th}$ element on the main diagonal of the covariance matrix Σ. The equations above update the prior and noise precision according to the posterior distribution. With the updated parameters, the posterior is refined, leading to an iterative algorithm for accurate posterior estimation by setting a converge criteria. The inferred row vector of GRN is obtained by the mean of estimated posterior distribution μ. The full inferred GRN matrix $\hat{A}$ is then constructed by stacking all inferred row vectors. In this paper, we simply set a maximum number of iterations to force the iteration to end, which is the only parameter BiGSM needs to adjust. We recommend setting this between 10 and 50 according to the size of the network, and in subsequent experiments the number of iterations was set to 35.

**Figure 1.**
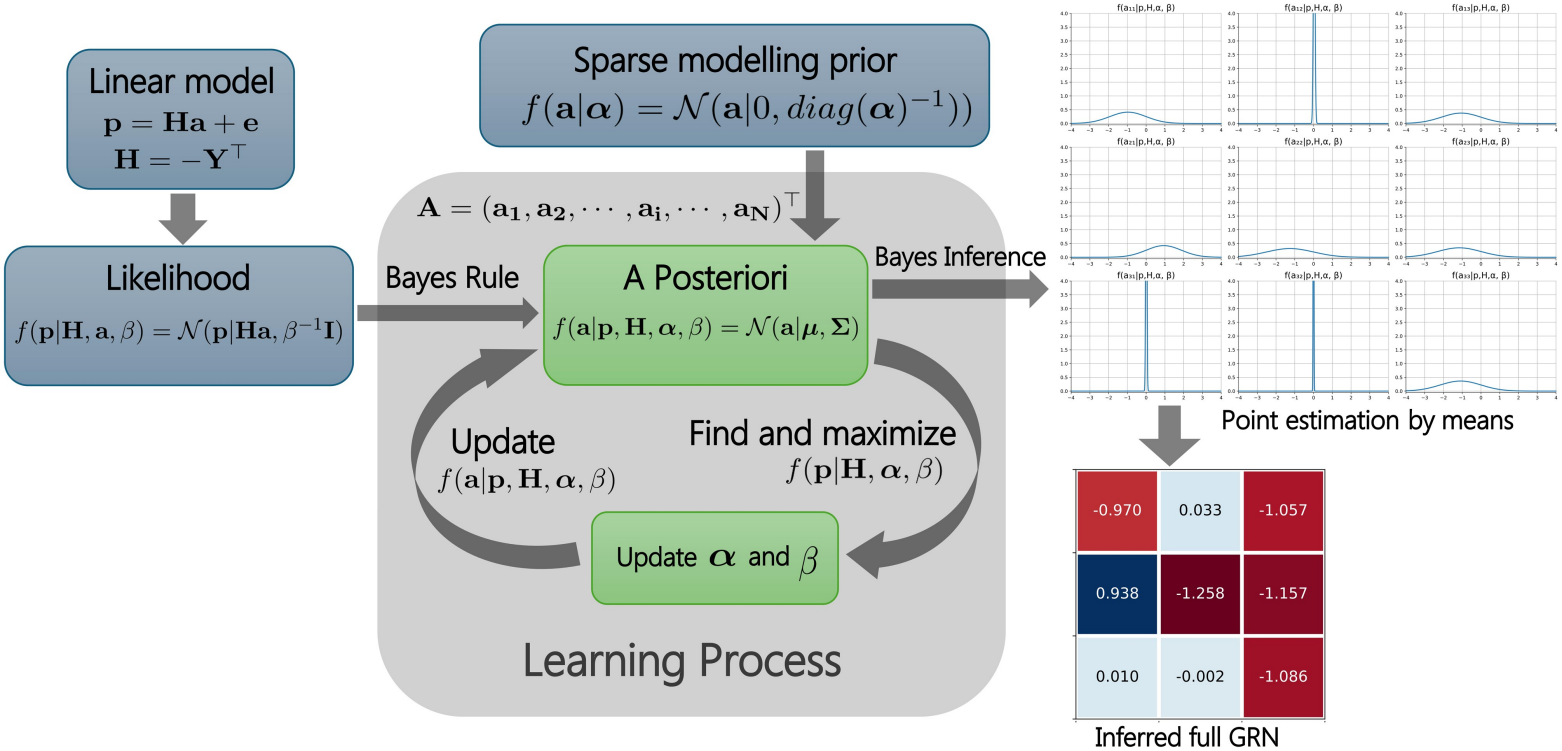
Overall workflow of the BiGSM algorithm. From the model assumption, the likelihood is computed. The posterior distribution is derived from Bayes rule. Then, the log-likelihood as a function of prior parameters and noise variance is computed from the posterior. In the iterative learning process, the prior parameters and noise variance are first updated at each step, then the posterior is updated in iterations.

Figure 1 illustrates the overall workflow of BiGSM. BiGSM takes the gene expression Y and perturbation P as inputs, and provides both point estimates and closed-form posterior distributions associated with each potential link in the inferred GRN. Note that BiGSM is a machine learning method, where the set of parameters is determined using type-2 maximum likelihood based learning method, also known as the evidence approximation (Bishop and Nasrabadi 2006). The ‘learning’ in BiGSM is only based on the given observation (gene expression) and perturbation data, and does not use any labelled dataset.

### 2.3 GRN inference in GeneSPIDER

To benchmark BiGSM against other state-of-the-art methods, we utilized the GeneSPIDER MATLAB toolbox (Tjärnberg *et al.* 2017, Garbulowski *et al.* 2024) to conduct experiments. This toolbox enables both GRN-based data simulation as fold-changes and GRN inference, making it a suitable benchmarking environment. We employed methods that demonstrated high performance in previous studies. These include “**P**-based” approaches that use the knowledge of perturbation design such as LSCON, LASSO, SVM and Zscore, which utilize both Y and P, as well as ‘non **P**-based’ method GENIE3, which relies solely on Y. In the context of GRN inference, all methods are unsupervised as none of them require a true GRN. Some ‘**P**-based’ methods such as LSCON, LASSO and SVM essentially require input-response variable pairs to estimate the fitted coefficients. For these methods, Y is the independent (input) variable and P is the dependent (response) variable, while the fitted coefficients are used as output edge weights in the estimated GRN, where their sign indicating activation (positive) or inhibition (negative). Here, LASSO and SVM iteratively utilize P. The Z-score method estimates edge weights and sign through a z-score-based transformation of Y and P. GENIE3 assigns edge weights based on link importance derived from a tree ensemble, producing an unsigned GRN. Finally, regarding the edge directions in the inferred GRN, we assume that they are considered from columns to rows of adjacency matrix for all methods including BiGSM.

We utilized the evaluation metrics provided by GeneSPIDER, including Area under the precision-recall (AUPR) curve, Area Under the Receiver Operating Characteristic (AUROC) curve, and maximum F1-score. Specifically, a range of threshold values is applied to each inferred GRN to enable comprehensive analysis over different sparsity levels, which converts the inferred GRN matrix into binary network for subsequent evaluation using the evaluation metrics.

## 3 Results

The effectiveness of the BiGSM algorithm was evaluated in three key areas: (i) Accuracy: the ability to infer GRN accurately under various conditions, (ii) Closed-form posterior distributions: the ability to obtain point estimates as well as posterior distributions of inferred GRN using the Bayesian method, and (iii) Density modelling: the ability to model the true GRN density and predict the weights correctly.

The benchmarking test for evaluating accuracy was mainly conducted on GeneSPIDER, DREAM, and GRNbenchmark datasets. To assess the posterior distributions computed by BiGSM, we generated a small GRN with GeneSPIDER and visualized the posterior distributions of the predicted GRN. Additionally, to showcase that BiGSM can capture the weights of GRN links, we compared the densities of a true GRN and inferred full GRNs from various methods, and highlighted that BiGSM effectively models the density of the true GRN.

### 3.1 Benchmarking on GeneSPIDER datasets

**Figure 2.**
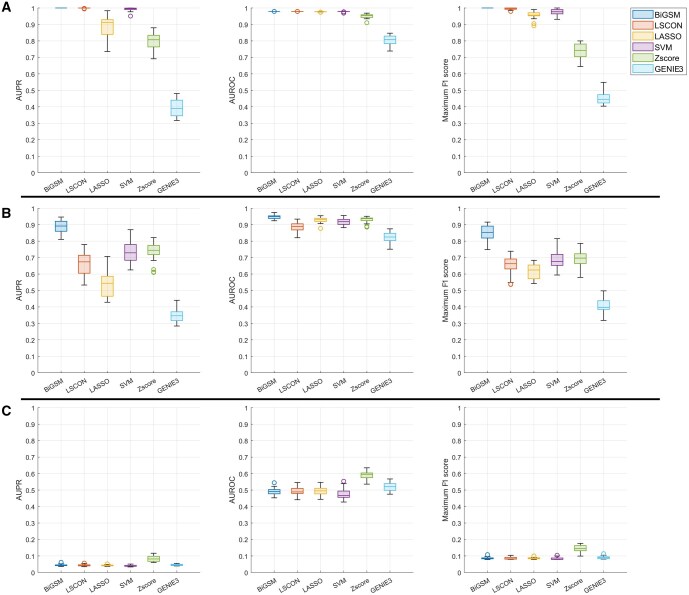
AUPR, AUROC, and Maximum F1 score of six inference methods on GeneSPDER data with SNR of 1 (A), 0.1 (B), 0.01 (C). The simulated data has one replicate and 50 genes for each GRN. The evaluation is without self-loops. Each box contains inference results over 20 GRNs.

**Figure 3.**
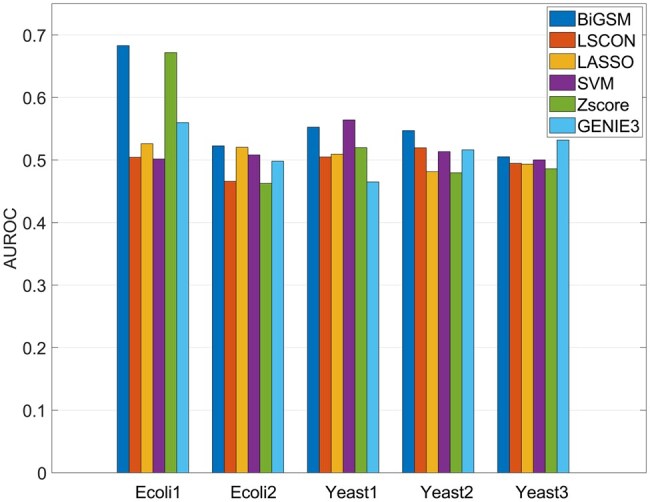
AUROC of six inference methods on DREAM4 Insilico size 100 networks, knockdown data. Each group of bars shows the AUROC of six methods on each network.

To evaluate the accuracy of predicted GRNs, we calculated AUPR, AUROC, and maximum F1 score on the GeneSPIDER datasets Fig. 2. The expression data was simulated under varying SNRs of 1, 0.1, and 0.01 and contained one replicate. During the evaluation, to examine the ability to discover complex interactions between genes, we did not include self-loops. More benchmark results on GeneSPIDER for the scenarios with self-loops and with three replicates can be found in the Figs 1–3, available as supplementary data at *Bioinformatics* online. Regarding the three accuracy metrics, BiGSM achieved the best performance at SNR 1 and 0.1. At SNR 1, BiGSM, LSCON, and SVM achieved excellent results. As the SNR dropped to 0.1, most methods experienced a decline in performance, but BiGSM maintained the highest accuracy on all three metrics. At SNR 0.01, an extremely noisy scenario, none of the methods could generate reasonable predictions, and all methods’ performance were almost equally poor. When self-loops were included, the performance of all methods increased, as self-loops are generally easier to predict. However, BiGSM continued to demonstrate outstanding performance, similar to its results without self-loops. Across all testing scenarios on the GeneSPIDER datasets with different SNRs and the presence or absence of self-loops, BiGSM showed the best overall performance among the six GRN inference methods.

### 3.2 Benchmarking on DREAM datasets

**Figure 4.**
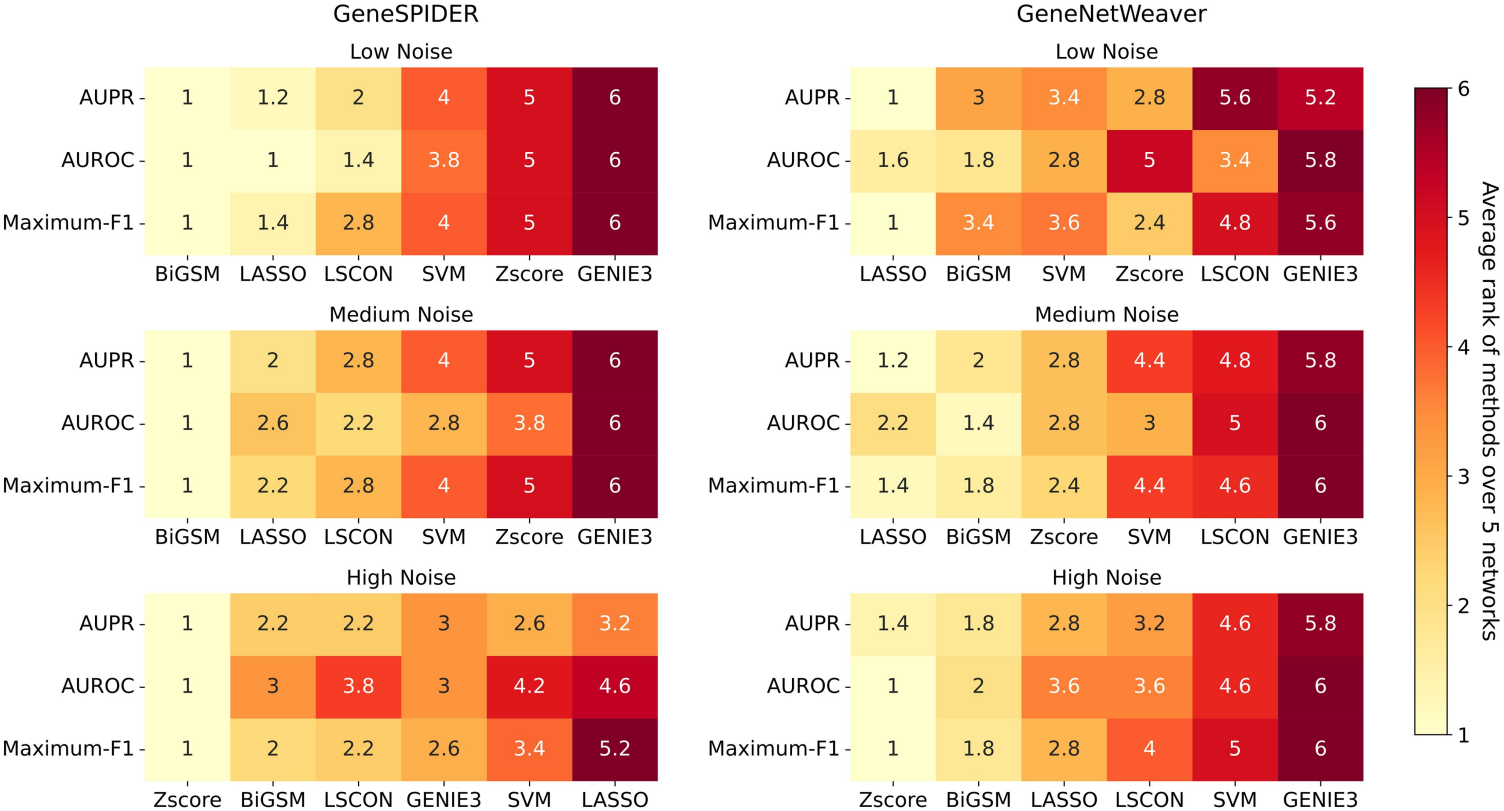
Average ranks of six inference methods on GRNbenchmark. Each number represents the average rank of the method across five networks using the corresponding evaluation metric and under the specific noise level. BiGSM has the best overall performance in the 6 challenges, with a summed ranking of 10.

**Figure 5.**
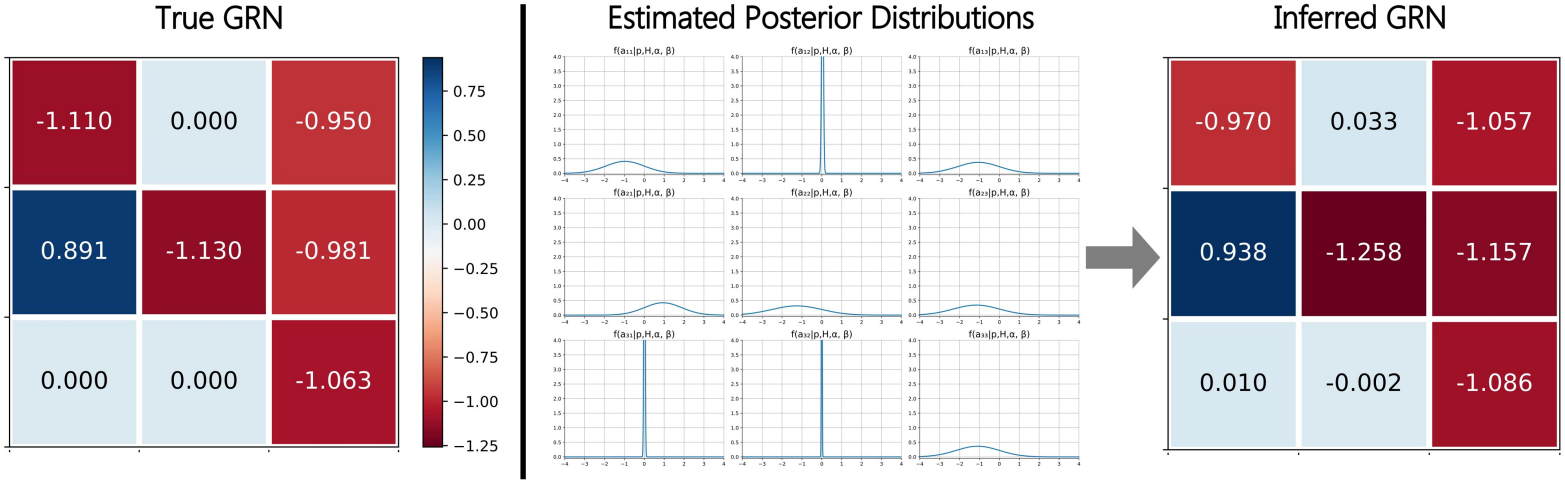
Estimated posterior distribution of a 3×3 GRN. The true GRN weights and the corresponding gene expression are generated by GeneSPIDER with SNR=0.1. The estimated posterior distributions are the probability density functions of predicted GRN weights. The inferred GRN are the mean values taken from posteriors.

To ensure that the performance of BiGSM is not biased for specific datasets, additional benchmarking tests were conducted on the DREAM challenge datasets simulated by GeneNetWeaver. AUROC was calculated for all methods on DREAM3, DREAM4, and DREAM5 datasets. On the DREAM4 size 100 knockdown data, BiGSM achieved the highest AUROC for three out of five networks (Net1, Net2, and Net4), only slightly trailing behind SVM on Net3 and GENIE3 on Net5 (Fig. 3). This indicates that BiGSM can outperform other inference methods across various datasets, even when these datasets are generated using different models. The AUROC on DREAM3 and DREAM5 are shown in Figs 4 and 5, available as supplementary data at *Bioinformatics* online, respectively, where BiGSM also showed competitive performance against other methods. On the DREAM3 dataset, BiGSM achieved the highest AUROC on the *E. coli* 1 and *E. coli* 2 networks and attained the second-highest AUROC on the Yeast 2 network. On the DREAM5 dataset, due to the low data availability, BiGSM did not outperform all the other methods but still surpassed the performance of SVM and Zscore, which showed great accuracy in high noise level data in GeneSPIDER. In general, although BiGSM faced challenges due to model mismatches, it still demonstrated good generalization across various datasets. This highlights BiGSM’s robustness and adaptability, despite the differences in data generation methods.

### 3.3 Benchmarking on the GRNbenchmark web server

**Figure 6.**
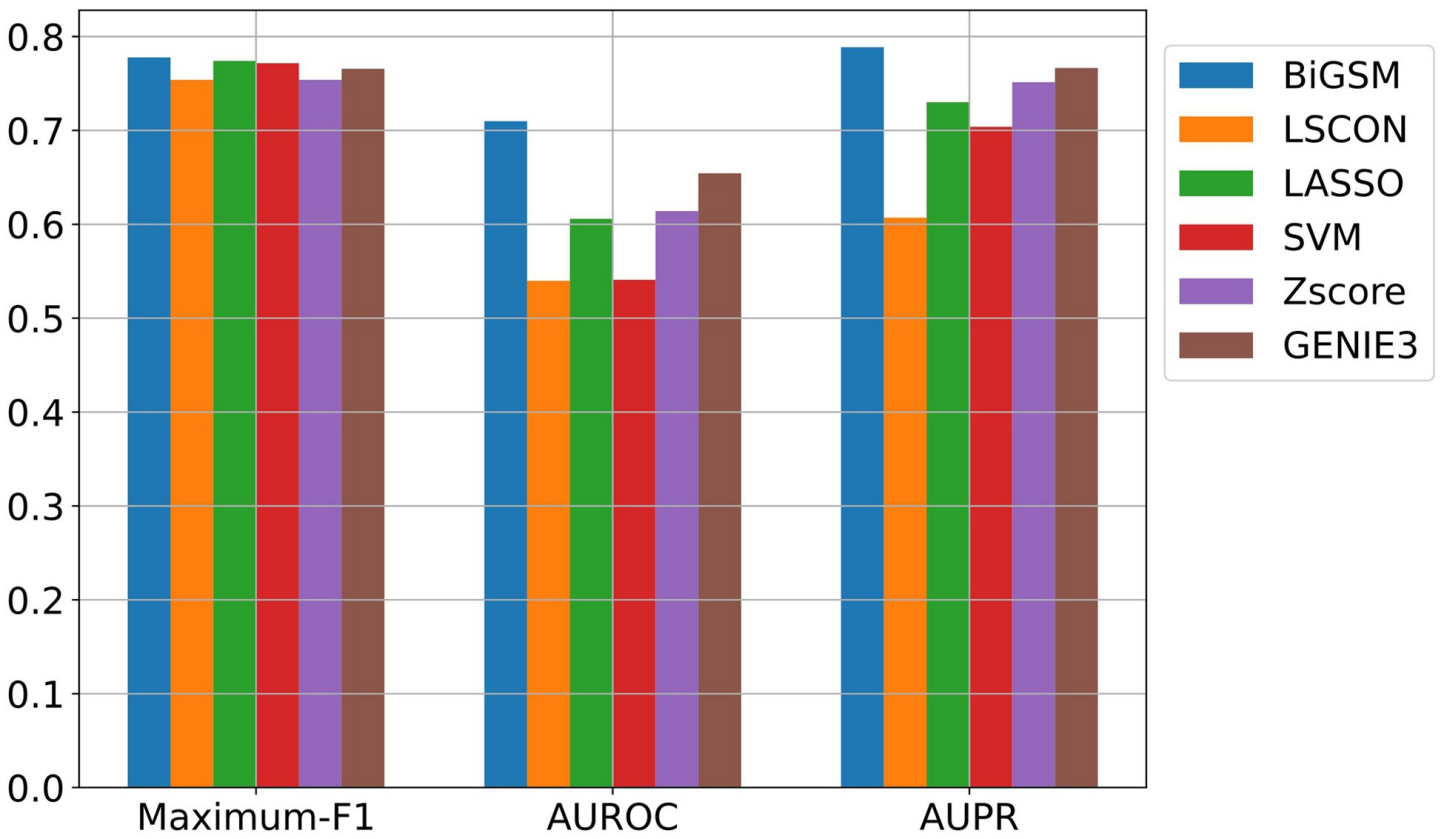
AUPR, AUROC, and Maximum F1 score of six inference methods on a *E. coli* network using biological expression data.

**Figure 7.**
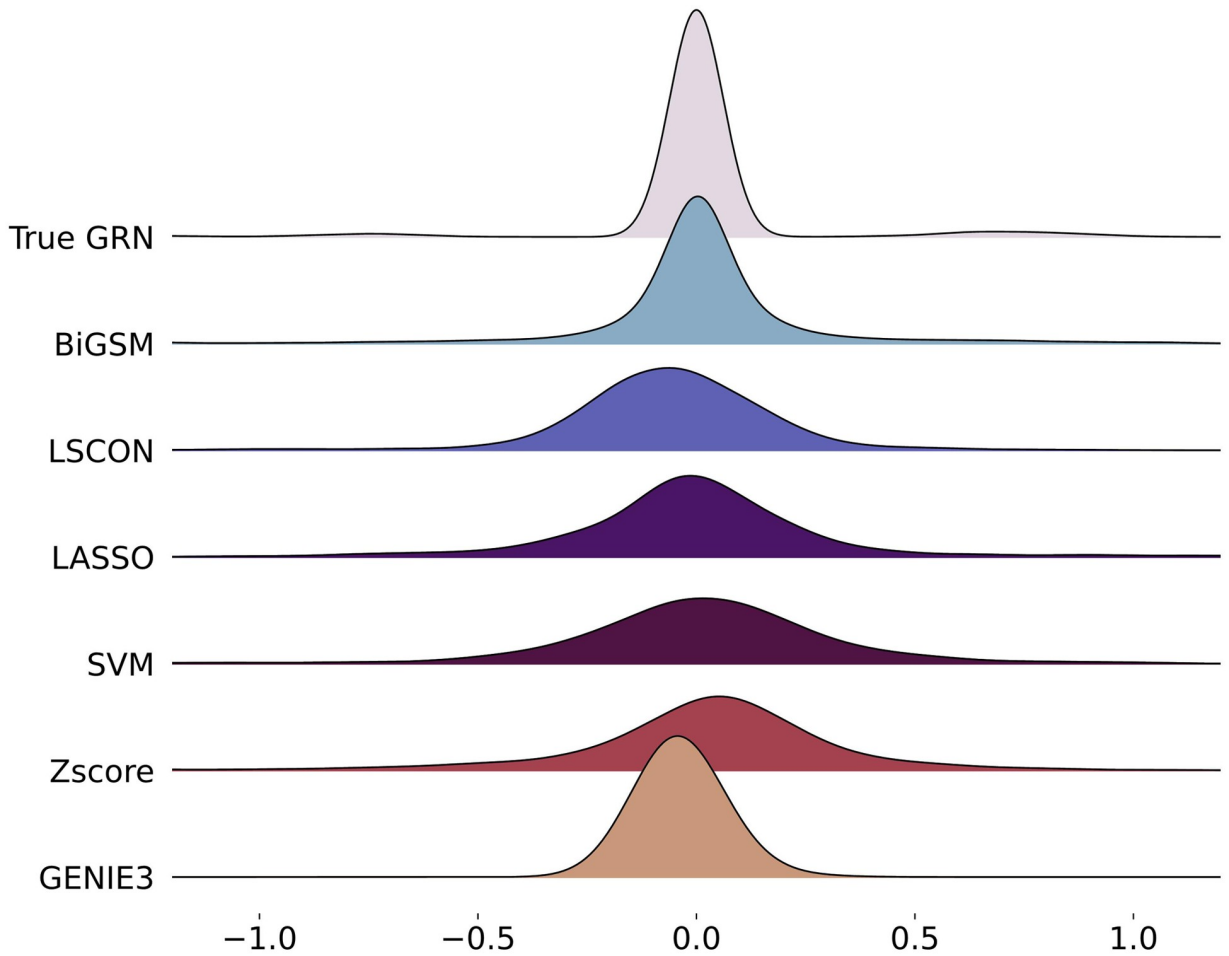
Analysis of density of inferred full GRNs over six methods. A kernel density estimation (KDE) with Gaussian kernel is used to estimate the probability density function (PDF) of the true GRN and the inferred GRNs.

To ensure the evaluation is fair and transparent, we evaluated BiGSM using a publicly available web server GRNbenchmark.org, which provides a complete benchmark pipeline. GRNbenchmark has 5 networks generated by GeneSPIDER and 5 networks generated by GeneNetWeaver. The expression data generated under three noise levels are provided for the GRN inference. GRNbenchmark calculates the AUPR, AUROC, and Maximum F1-score and directly displays the results on the website. Figures 6 and 7, available as supplementary data at *Bioinformatics* online illustrates the results provided by GRNbenchmark. These two scatters plots in the supplementary use the results saved from GRNbenchmark.org directly. The complete results, including underlying curves and tables with more details, can be found at GRNbenchmark.org.

To better visualize the results on GRNbenchmark and compare the performance of different methods, we ranked the methods by their performance for each network, each noise level, each evaluation metric, and each data group. Then, we calculated the average rank and sorted the methods according to this. To ensure the consistency of the benchmark test, we only ranked the methods that were compared before. Figure 4 shows the average ranks for all methods over 5 networks in each case. The performance of BiGSM is consistent with our previous findings. BiGSM performed excellently and ranked first among all methods under low and medium noise conditions on GeneSPIDER data, aligning with our prior results. In high-noise conditions, most methods, including BiGSM, struggled due to severe noise interference. Only the Zscore method managed to achieve reasonable results. This indicates that BiGSM, like many other methods, has limitations when handling extremely noisy data. On GeneNetWeaver data, BiGSM demonstrated competitive performance and provided reliable inferences across various noise levels, even under high noise conditions. Across three noise conditions, BiGSM achieved the second-highest average rank in three evaluation metrics. Although BiGSM is not the best method in all 6 challenges, ranking behind LASSO and Zscore in some cases, it still has the best overall performance: comparing the sum of ranks in six challenges, BiGSM reaches an overall rank of 10, while the runner-up method LASSO reaches 15 and Zsocre gets 19. This suggests that BiGSM is an efficient method for GRN inference. It can deliver reliable and accurate predictions, particularly in low and medium-noise environments, showing its potential as a valuable tool in GRNs analysis. In addition, it can provide variance between its predictions as posterior distributions, showing the confidence of the predicted weights, explained in Subsection 3.4.

### 3.4 Posterior distribution found by Bayesian inference

Figure 5 shows how our BiGSM found the posterior distribution for a 3 by 3 toy GRN matrix. By incorporating a sparse modelling prior distribution for each potential link, BiGSM provides a closed-form posterior for every link in the network. As shown in the figure, links that are likely to be zero exhibit sharp shapes in posterior distributions, with mean values close to zero. This indicates that the model automatically identifies zero entries, assigning them high confidence as the sparse positions. Conversely, the posterior distributions for the non-zero entries are more flat, which means the possible values for these entries are more diverse. BiGSM chooses the mean values of posterior distribution as a point estimation, which are the values with the highest posterior probability. This example demonstrates that BiGSM not only delivers precise point estimates but also uncovers the complete posterior distribution for all possible links, providing an informative view of the GRN’s structure.

### 3.5 GRN inference on biological data

To evaluate the inference performance on real biological expression data, BiGSM was tested on a sub-network of the SOS path way in *E. coli* (Gardner *et al.* 2003) with nine genes. We compared its performance against the same set of GRN inference methods in Fig. 6, where BiGSM achieved the highest Maximun F1 score, showing its ability to provide the most accurate inference at optimal sparsity. BiGSM also obtained the highest AUROC and the highest AUPR, making it be the top performer among six methods on this biological dataset.

### 3.6 Density analysis

To further evaluate the inference accuracy on the weight of links, in this section, we analysed the densities of inferred GRNs from six inference methods and the true GRN. Figure 7 compares the densities of a true GRN and six inferred GRNs. The true GRN is generated by GeneSPIDER and consists of 50 genes with an average sparsity of five links per node. The expression data is generated with an SNR of 0.1, using only one replicate. The predicted GRNs are min-max normalized using the min and max values from the true GRN and shifted to have the original mean value before normalization. The predicted GRN by BiGSM has the most similar density to the true GRN, indicating that BiGSM can not only predict the existence of links but also calculate the weights of links accurately. Since the predicted weights from BiGSM are simply the means of posterior distribution, this result shows the effectiveness of BiGSM’s Bayesian inference approach which uses a probability model and maximum-likelihood estimation for each possible link.

### 3.7 Computational complexity analysis and scalability

**Figure 8.**
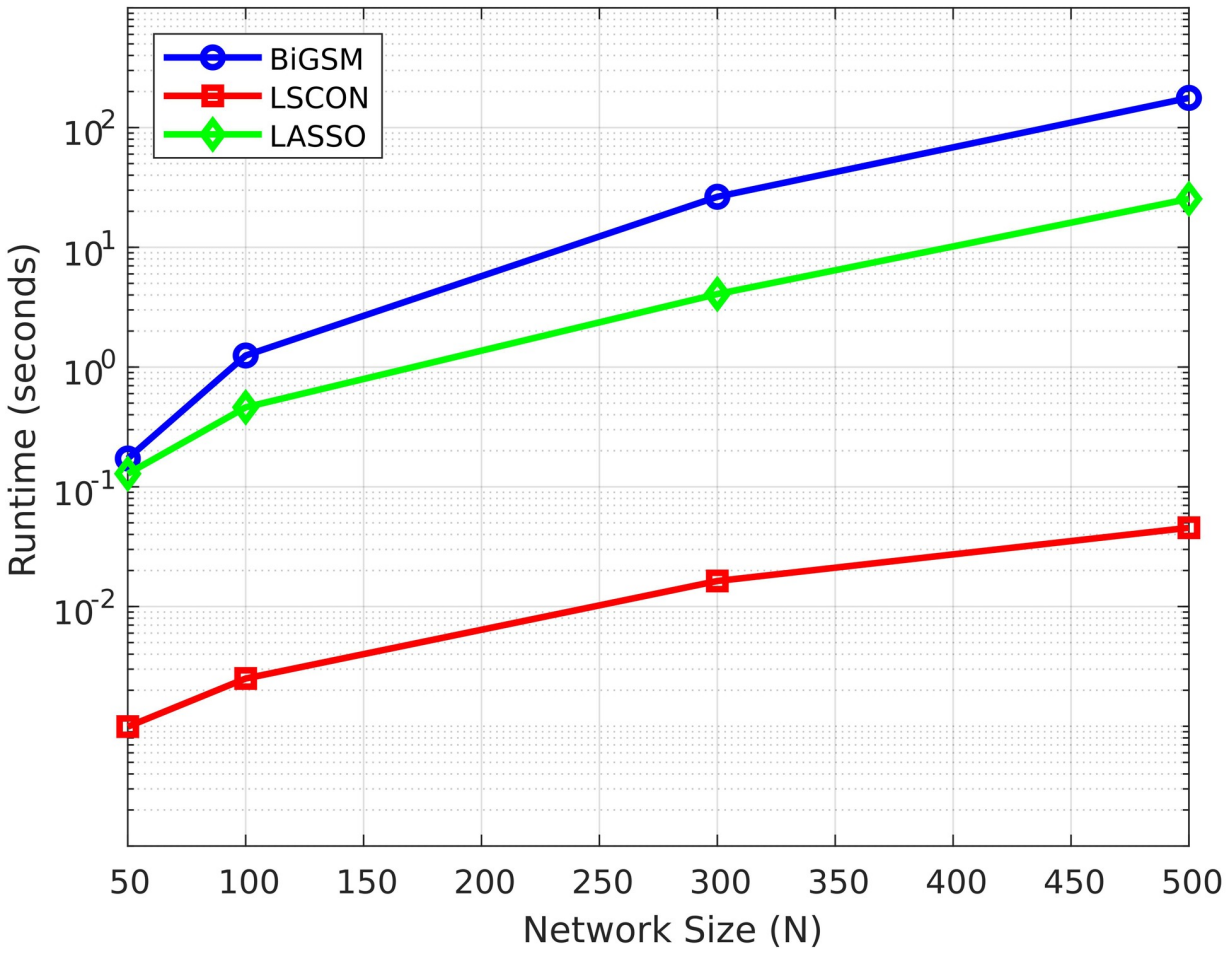
Average execution time of BiGSM, LSCON, and LASSO for varying number of genes (network size *N*) using standard tic-toc function.

We analysed the computational complexity of BiGSM by reporting the mean execution time over ten instances with varying number of genes using GeneSPIDER toolbox, and the results are shown in Fig. 8 using standard tic-toc function. The expression matrix has the same size as the network which is N×N. We compared BiGSM with LSCON and LASSO since they are all based on the same model assumption and estimating the GRN matrix directly. When the network size is small (N≤50), BiGSM requires similar execution time as LASSO. As the network size increases, BiGSM is around ten times slower than LASSO. This is because BiGSM incorporates an iterative scheme. Naturally the required execution time will be longer if the number of genes is high.

## 4 Discussion and conclusions

We presented BiGSM, a novel GRN inference algorithm based on Bayesian inference and sparsity modelling. BiGSM innovatively utilizes the sparsity of the GRN adjacency matrix and considers GRN inference as a sparse matrix estimation problem, which is a well-studied domain in statistical signal processing. Within a widely used GRN system model, we derived how to obtain the closed-form posterior distributions of an inferred GRN from expression data and perturbation matrix by selecting suitable prior distributions and the maximum likelihood learning.

Our benchmarking test showed that BiGSM performs consistently well at various noise levels and remains competitive in datasets from different data generation models to real biological data. Other GRN inference methods are usually sensitive to noise levels and no method performs best at all noise levels, for example, LSCON achieved highly accurate inference performance in low noise, but the accuracy dropped massively when the noise level increase. Zscore and handled noisy data very well but it was less accurate than most of the methods when the noise level is low. On the contrary, BiGSM was shown to be fairly robust in terms of noise levels and achieve reliably high accuracy in most cases. In addition, BiGSM calculates the posterior distributions of the inferred GRN, which allows link selection based on the inferred probabilities. Some other methods can also provide edge confidence, for example, GENIE3 provides ranking for edges, and LASSO with bootstrap or stability selection framework can also provide edge confidence, but these are not based on the posterior distribution. In BiGSM, links predicted to be non-existent and existent have easily distinguishable features in their posterior distributions, suggesting that the sparse modelling of BiGSM is effective. The analysis of inferred GRNs’ densities also showed that BiGSM could reconstruct the original GRN density, which was in line with its high accuracy.

In our benchmark, most methods rely on fold-change bulk RNA-seq data, where all genes were perturbed. In this setting, it is crucial to indicate whether a gene is knocked down by using fold-change values, typically resulting in highly negative expression levels. This approach has demonstrated high performance in our previous studies Seçilmiş *et al.* (2022a,b). However, all methods, including BiGSM, can also be applied to perturbed single-cell RNA-seq data for GRN inference (Replogle *et al.* 2020).

To ensure consistency and fairness in validation, we primarily benchmarked BiGSM against other regression methods, as they all directly estimate the weights of the GRN matrix. Other methods based on correlation scores [e.g. MI-based CN (Aghdam *et al.* 2015), OIPCQ (Mahmoodi *et al.* 2021), and PCA-CMI (Zhang *et al.* 2012)] focus on predicting the strength regulations, but they do not directly estimate the GRN matrix, and they are not used for a direct comparison in this article. Future studies could explore integrating these complementary approaches for improved GRN inference.

In conclusion, we have shown that BiGSM is an accurate and robust method that outperforms many published GRN inference methods on varying noise levels and datasets. Incorporation of prior knowledge is an effective way to improve the inference performance (Werhli and Husmeier 2007, Greenfield *et al.* 2013), and BiGSM incorporates a significant prior knowledge that the GRN matrix is sparse, and designs the inference approach based on it specifically. As a Bayesian method, BiGSM outputs the posterior distributions of the predictive network in addition to accurate point estimates, providing more valuable information than other methods. We believe that our method can support the inference of GRNs in cancer research and for other complex diseases.

## Supplementary Material

btaf318_Supplementary_Data

## Data Availability

The data underlying this article are available in ZENODO, at https://doi.org/10.5281/zenodo.13940397. The data and code are also available at https://github.com/SachLab/BiGSM.
