# Supplementary material for: BiGSM: Bayesian inference of gene regulatory network via sparse modelling
Source: Bioinformatics. 2025 Jun 9;41(6):btaf318. doi: 10.1093/bioinformatics/btaf318 (PMC12151459; doi:10.1093/bioinformatics/btaf318)
Supplement: btaf318_Supplementary_Data [file btaf318_supplementary_data.zip › Supplementary material online.pdf]

Supplementary material for "BiGSM: Bayesian inference of Gene Regulatory Network via sparse modelling".

1 Summary of datasets used

A summary of the dataset used is given by Table S1. N_{gene} stands for average number of genes, N_{TF} is the average number of TFs, S is the average sparsity represented by average number of edges per gene, N_{sample} is the number of samples, 'simulation' is whether the data is simulated or real biological, where 'True' means both GRN and expression are simulated, 'Both' means some GRN are real but expression is simulated, 'False' means both GRN and expression are real biological. Since the true network of the GRNbenchmark data set is unavailable for the user, they are not provided here.

2 Benchmark results on GeneSPIDER

The following box charts show the benchmark results on GeneSPIDER data with other conditions and varying SNRs (1, 0.1, 0.01). Fig.S1 shows the results with 1 replicate and self-loops included. Fig.S2 shows the results with 3 replicates and self-loops excluded. Fig.S3 shows the results with 3 replicates and self-loops included. Each box contains inference results over 20 GRNs with 50 genes and the sparsity is 3 edges per gene on average. The expression data has one replicate.

3 Benchmark results on DREAM challenge data

Fig.S4 shows the AUROC of six methods on DREAM3 size 50 networks using expression data with knockout experiment. Fig.S5 shows the AUROC of six methods on DREAM5 with a selected subnetwork containing genes perturbed by single knockout and over-expression experiments.

4 Benchmark results on GRNbenchmark.org

Fig.S6 and Fig.S7 shows the original benchmark results of six methods with AUPR, AUROC and maximum F1 score on GRNbenchmark.org, using GeneSPIDER and GeneNetWeaver datasets respectively.

Table S1: Summary of datasets used.

Dataset	N_{gene}	N_{TF}	S	N_{sample}	Simulation
GeneSPIDER	50	50	3	120	True
DREAM3	50	26	2.2	5	Both
DREAM4	100	39	2	5	Both
DREAM5	41	20	0.69	2	Both
GRNbenchmark	100	-	-	30	True
E.coli	9	9	5.4	1	False

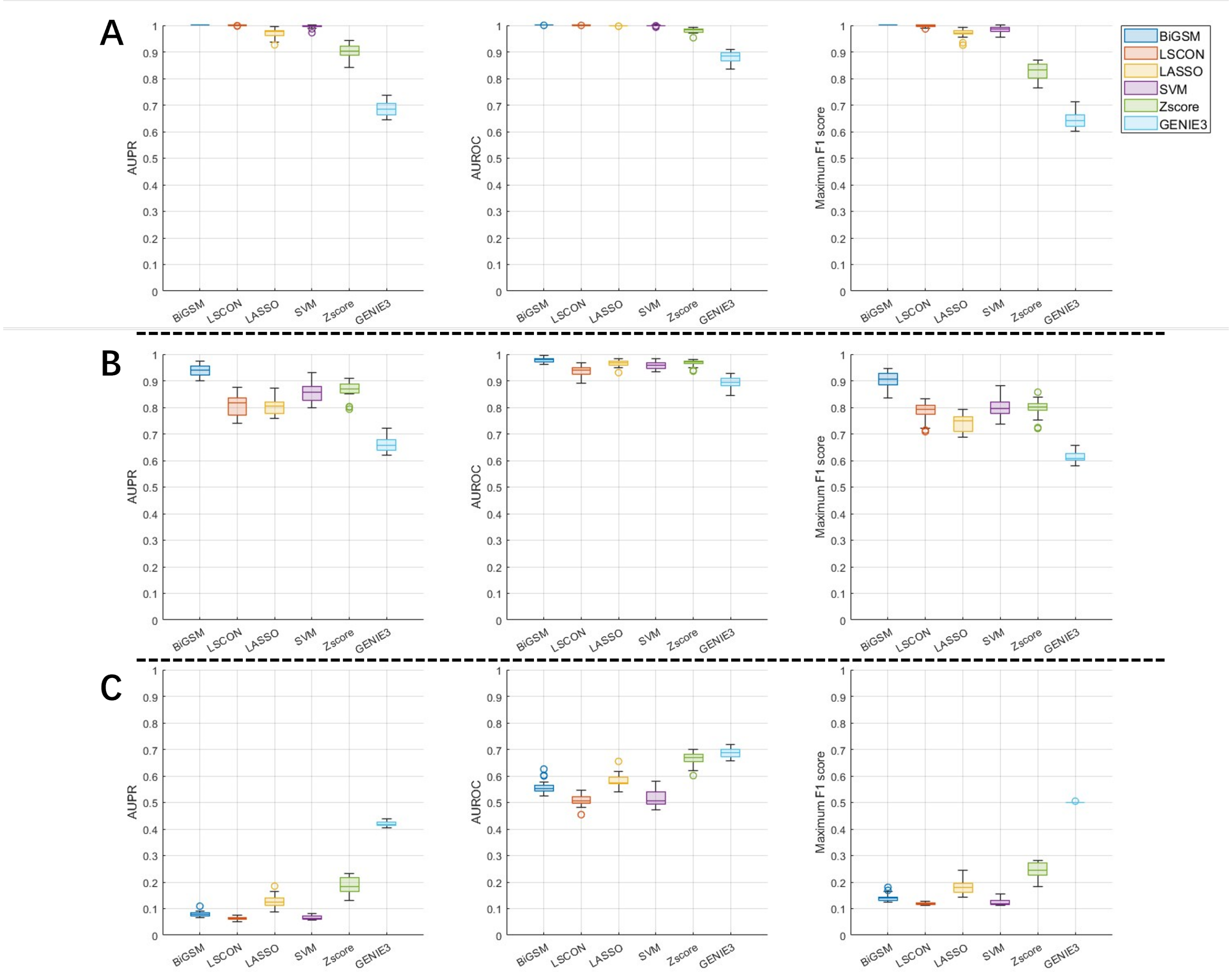

Figure S1: AUPR, AUROC, and Maximum F1 score of six inference methods on GeneSPDER data with SNR of 1 (A), 0.1 (B), 0.01 (C). The simulated data has 1 replicate and 50 genes for each GRN. The evaluation included self-loops. Each box contains inference results over 20 GRNs.

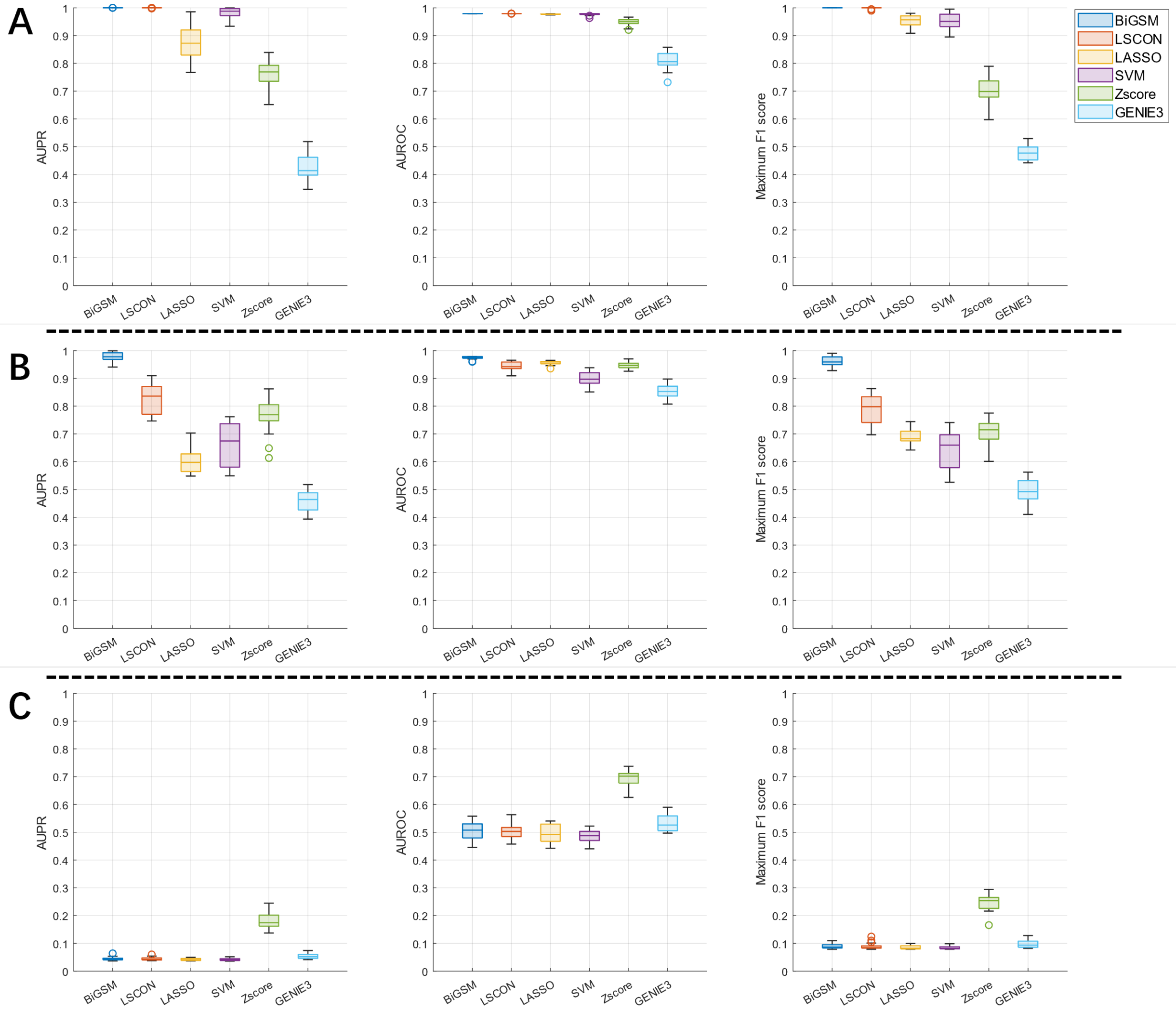

Figure S2: AUPR, AUROC, and Maximum F1 score of six inference methods on GeneSPDER data with SNR of 1 (A), 0.1 (B), 0.01 (C). The simulated data has 3 replicate and 50 genes for each GRN. The evaluation is without self-loops. Each box contains inference results over 20 GRNs.

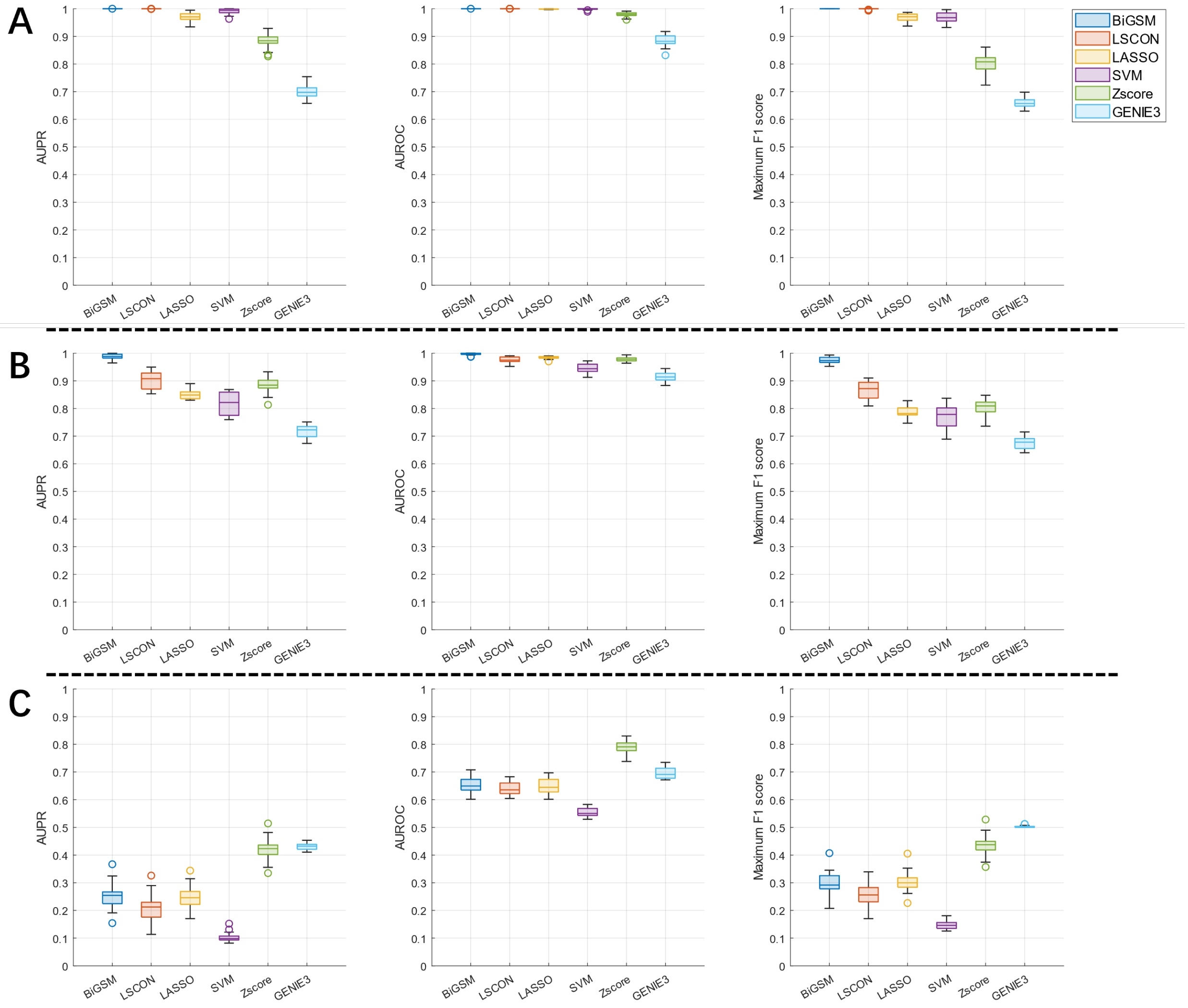

Figure S3: AUPR, AUROC, and Maximum F1 score of six inference methods on GeneSPDER data with SNR of 1 (A), 0.1 (B), 0.01 (C). The simulated data has 3 replicate and 50 genes for each GRN. The evaluation included self-loops. Each box contains inference results over 20 GRNs.

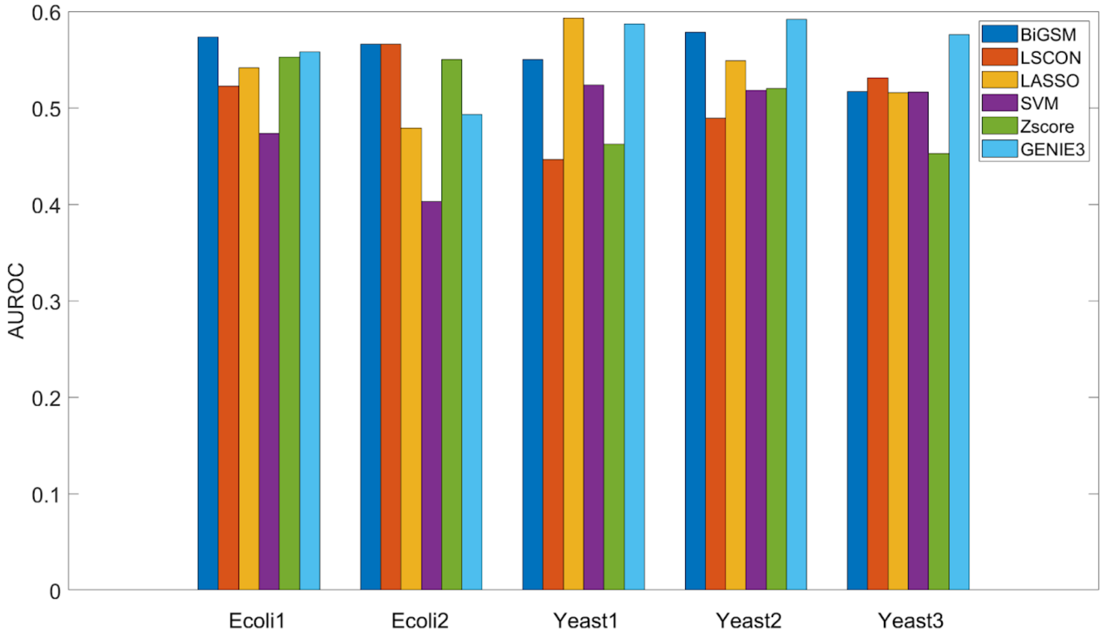

Figure S4: AUROC of six inference methods on DREAM3 Insilico size 50 networks, knockout data. Each group of bars shows the AUROC of six methods on each network.

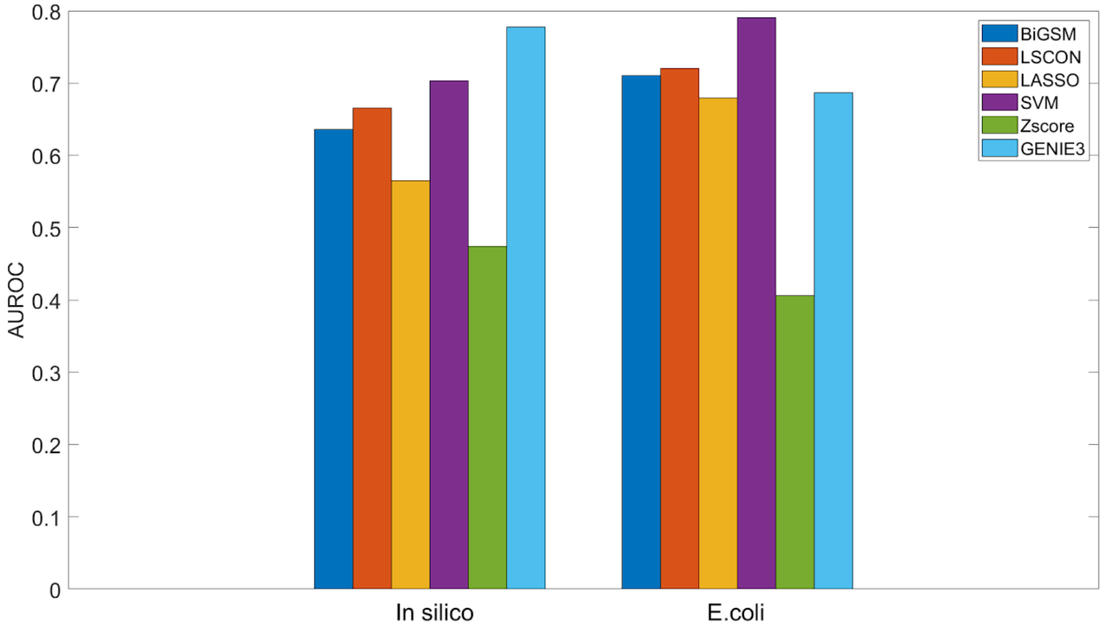

Figure S5: AUROC results from the analyses on the subsets of the DREAM5 in silico and E. coli datasets.

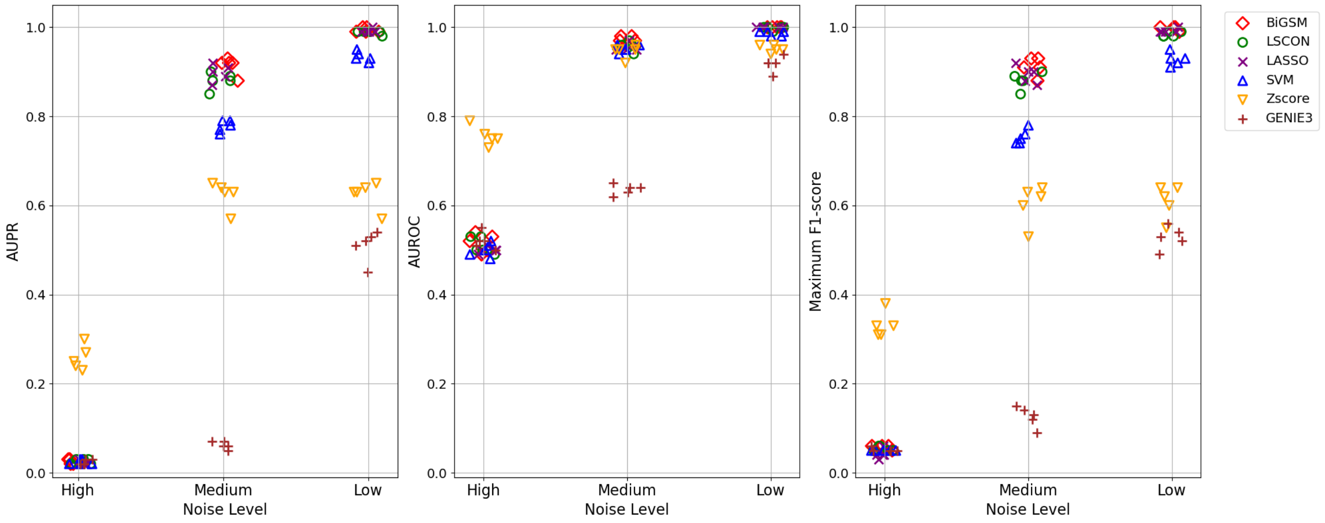

Figure S6: Scatter plots with original benchmark results on GRNbenchmark webserver, GeneSPIDER dataset.

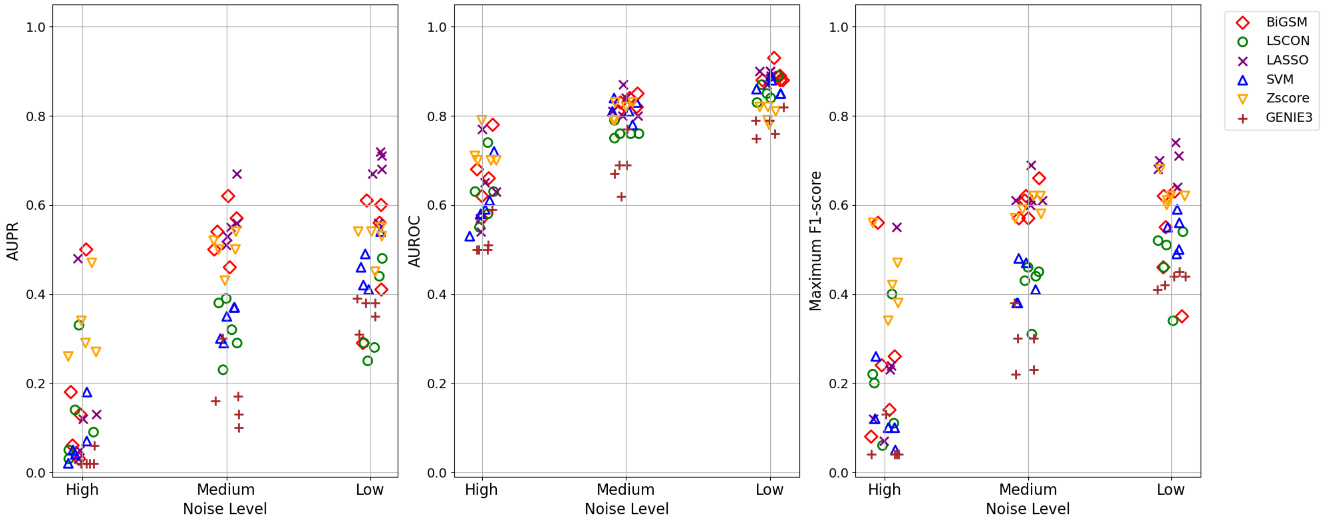

Figure S7: Scatter plots with original benchmark results on GRNbenchmark webserver, GeneNetWeaver dataset.
